# Characterization of the interactions of PARP-1 with UV-damaged DNA *in vivo* and *in vitro*

**DOI:** 10.1038/srep19020

**Published:** 2016-01-12

**Authors:** Nupur K. Purohit, Mihaela Robu, Rashmi G. Shah, Nicholas E. Geacintov, Girish M. Shah

**Affiliations:** 1 Laboratory for Skin Cancer Research, CHU-Q (CHUL) Quebec University Hospital Research Centre & Laval University, 2705, Laurier Boulevard, Québec (QC) Canada G1V 4G2; 2 New York University, Department of Chemistry, New York, NY, USA

## Abstract

The existing methodologies for studying robust responses of poly (ADP-ribose) polymerase-1 (PARP-1) to DNA damage with strand breaks are often not suitable for examining its subtle responses to altered DNA without strand breaks, such as UV-damaged DNA. Here we describe two novel assays with which we characterized the interaction of PARP-1 with UV-damaged DNA *in vivo* and *in vitro*. Using an *in situ* fractionation technique to selectively remove free PARP-1 while retaining the DNA-bound PARP-1, we demonstrate a direct recruitment of the endogenous or exogenous PARP-1 to the UV-lesion site *in vivo* after local irradiation. In addition, using the model oligonucleotides with single UV lesion surrounded by multiple restriction enzyme sites, we demonstrate *in vitro* that DDB2 and PARP-1 can simultaneously bind to UV-damaged DNA and that PARP-1 casts a bilateral asymmetric footprint from −12 to +9 nucleotides on either side of the UV-lesion. These techniques will permit characterization of different roles of PARP-1 in the repair of UV-damaged DNA and also allow the study of normal housekeeping roles of PARP-1 with undamaged DNA.

The abundance of poly (ADP-ribose) polymerase-1 (PARP-1) in mammalian cells and its rapid catalytic activation to form polymers of ADP-ribose (PAR) in the presence of various types of DNA damages with or without strand breaks has made it an ideal first responder at the lesion site to influence downstream events^1,2^. Apart from DNA damages, PARP-1 is also recruited to DNA during normal physiological processes such as transcription and chromatin remodeling^3^, which do not involve overt DNA damage but just altered DNA structures. While we know much more about how PARP-1 rapidly recognizes and binds to single or double strand breaks in DNA, we know very little about how PARP-1 interacts with DNA damages or altered DNA structures without strand breaks. The key reason is that the existing methodologies that readily identify interactions of PARP-1 with DNA strand breaks are not sufficiently sensitive to study the relatively weaker responses of PARP-1 to DNA damage without strand breaks. The response of PARP-1 to UVC-induced direct photolesions, such as cyclobutane pyrimidine dimers (CPD) that are formed without any DNA strand breaks exemplifies this problem.

Recent studies from others and our team have shown the involvement of PARP-1 in the host cell reactivation^4^ and specifically in the nucleotide excision repair (NER) of UV-damaged DNA through its interaction with early NER protein DDB2 ^5–7^. Additional studies have shown that downstream NER proteins XPA^8,9^ and XPC^10^ are PARylated. Thus, PARP-1 possibly has multiple roles in NER, but we do not yet fully understand its interactions with UV-damaged DNA or other NER proteins due to two major challenges. The first challenge is that unlike for many NER proteins, the abundance of endogenous PARP-1 in the nucleus makes it nearly impossible to visualize its dynamics of recruitment to UV-damaged DNA *in situ* using conventional immunocytological methods. To circumvent this challenge, the detection of its activation product PAR has been used as a proxy for PARP-1 recruitment at UV-lesion^5,11^. However, PAR may underestimate the role of PARP-1 in response to UV-damage due to weak activation of PARP-1 by UV^4,12^, short half-life of PAR^2^, and technical limitations in combining the detection of PAR with other proteins^13,14^. PAR detection will also not reveal participation of PARP-1 in protein-protein interactions without formation of PAR. Thus, there is a need for methods that permit direct visualization of recruitment of PARP-1 to UV-induced DNA lesions.

The second major challenge is that we do not know the exact footprint of PARP-1 at the UV-lesion site that could explain its interaction with different NER proteins. We have earlier shown that PARP-1 binds to UV-damaged large oligonucleotide *in vitro* or to chromatin fragments containing T-T lesions *in vivo*^11^. We also showed that PARP-1 and DDB2 associate with each other on the chromatin of UV-irradiated cells and that DDB2 stimulates catalytic activity of PARP-1 in the presence of UV-damaged DNA^7^. However, these assays lack the nucleotide level resolution to reveal whether PARP-1 was bound directly to the UV-damaged bases or to any other base in those long pieces of DNA and whether PARP-1 and DDB2 have sufficient space to co-exist around UV-induced DNA lesion. To address these challenges, here, we describe two novel assays. The first assay is an *in situ* fractionation technique that allows a direct visualization of PARP-1 recruited to UV-damaged DNA *in vivo*. The second assay involves use of model oligonucleotides with a defined UV-damage surrounded by multiple restriction enzyme sites that reveals a bilateral asymmetric footprint of PARP-1 around the UV-lesion.

## Results and Discussion

### Novel *in situ* fractionation protocol to reveal recruitment of endogenous PARP-1 to UV-induced DNA lesion

**Figure 1:**
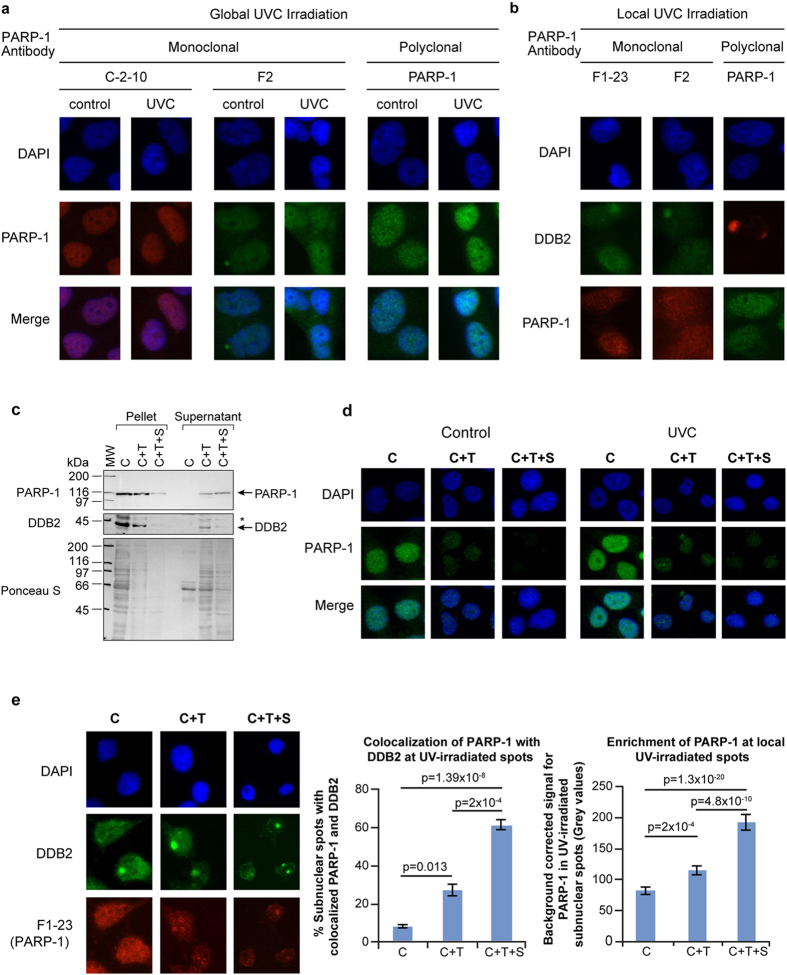
*In situ* fractionation to reveal the recruitment of endogenous PARP-1 to UV-induced DNA lesion site. (**a,b**) Unchanged pattern of nuclear staining for PARP-1 after global or local UVC-irradiation of cells processed with conventional immunocytological techniques. Human skin fibroblasts were exposed either to global (panel a) or local (panel b) irradiation with UVC, fixed with formaldehyde-methanol and probed for PARP-1 (global and local UVC) and DDB2 (local UVC) using specified antibodies. DAPI staining was carried out to define nuclei. **(c)** Efficiency of extraction of free PARP-1 and DDB2 from adherent control GMU6 cells. The pellets and supernatants obtained from equivalent cell numbers after extraction with CSK buffer (C), CSK+0.5% Triton (C+T) or CSK + 0.5% Triton + 0.42 M NaCl (C+T+S) were immunoblotted for PARP-1 and DDB2. The *refers to non-specific signal in DDB2 probing and Ponceau S staining reflected the residual protein content in cell pellets and supernatant at the end of each protocol. **(d)** Comparison of the efficiency of three protocols for extraction of the endogenous PARP-1 from adherent control and UV-irradiated cells. The GMU6 human skin fibroblasts were globally irradiated with 10 J/m^2^ UVC (or control), extracted 10 min later with the three protocols and probed for PARP-1 using polyclonal PARP-1 antibody. **(e)** Colocalization of endogenous PARP-1 with DDB2 at local UVC-induced DNA damage. GMU6 cells were irradiated locally with 100 J/m^2^ and after 10 min subjected to the three protocols (C, C+T and C+T+S) followed by visualization of PARP-1 (F1-23, red) and DDB2 (green). The left chart represents the percent of subnuclear PARP-1 spots that colocalize with DDB2. The right chart represents the quantification of the PARP-1 intensity at the DDB2 spots after subtraction of background signal intensity for PARP-1 from an equivalent area of unirradiated part of the same nucleus. Data of the charts are pooled from three experiments (*n* = 120–150 spots, data points are mean ± s.e.).

We first determined whether different permeabilization-fixation protocols conventionally used for PARP-1 could reveal a direct recruitment of PARP-1 to UVC-induced DNA photolesions *in situ*. There was no change in the pattern of abundant PARP-1 signal before or after global UVC-irradiation using formaldehyde-methanol protocol despite using three different antibodies to PARP-1 (Fig. 1a, left panel). Unlike global irradiation, local UVC-irradiation produces defined subnuclear spots of UV-damaged DNA that could be identified either by immunodetection of T-T lesions or DDB2 that is recruited very rapidly to these lesions (Supplementary Fig. S1a). Therefore, we examined whether the formaldehyde-methanol protocol could reveal localization of PARP-1 to subnuclear UV-damaged DNA spots after local irradiation. Once again, we could not observe colocalization of PARP-1 with the subnuclear spots of DDB2 (Fig. 1a, right panel). Next, we tried previously described formaldehyde-Triton protocol^8^ which was shown to display a punctate pattern of PARP-1. However, we noted that this pattern did not correlate with recruitment of PARP-1 to UV-damaged DNA, because it was observed in both the unirradiated control and globally UVC-irradiated cells; and none of the spots of PARP-1 were co-incident with DDB2, i.e., UV-damaged DNA in the cells after local UVC-irradiation (Supplementary Fig. S1b).

In view of these challenges in the immunocytological detection of PARP-1 bound to UV-damaged DNA due to the background “noise” created by rest of the nuclear PARP-1, we designed a novel *in situ* fractionation technique to selectively deplete unbound or “free” PARP-1 from the nuclei while leaving behind the PARP-1 that is bound and cross-linked to the UV-damaged DNA. We used CSK buffer (C) with Triton (C+T) as the basic conditions, which have been used earlier to extract majority of the cellular proteins without destroying the cellular architecture and permit visualization of NER and other repair proteins recruited to the damaged DNA^15–18^. To this buffer, we added 0.42 M NaCl (C+T+S), since we had earlier seen that 0.42 M NaCl retained chromatin-bound PARP-1 during cell fractionation *in vitro*^7^ whereas 1.6 M NaCl was shown to strip almost all PARP-1 from cells^19^. We first compared the efficiency of these three protocols (i.e., C, C+T and C+T+S) for the extraction of PARP-1 and DDB2 from unirradiated control cells. The immunoblotting of cell pellet and supernatant from each protocol revealed that while C+T protocol could efficiently remove majority of the free DDB2, a significant extraction of the free PARP-1 from cell pellet required C+T+S protocol (Fig. 1c). Next, we compared the capacity of these three protocols for the *in situ* extraction of PARP-1 from control and global UVC-irradiated cells. The immunocytological visualization confirmed that C+T+S buffer extracted most of the “free” PARP-1 from the control and UVC-irradiated cells, while leaving behind residual PARP-1 that would be interacting with DNA for normal physiological functions in control cells and relatively stronger punctate pattern of PARP-1 in UV-irradiated nuclei (Fig. 1d).

When the three protocols were compared after local irradiation, we observed that the C+T+S protocol offered the best extraction condition for visualization of the recruitment of PARP-1 to the subnuclear spots of DDB2 (Fig. 1e, left panel). The pooled data from at least 100 subnuclear spots revealed that each additional step of extraction with detergent and salt improved our ability to discern colocalization of PARP-1 with DDB2 (Fig. 1e, left chart). Since the initial irradiation conditions were identical prior to extraction with each of the protocols, the improved detection of colocalization of PARP-1 with DDB2 could only be due to a more efficient removal of rest of the nuclear “free PARP-1” by the C+T+S protocol. This was evident when PARP-1 signal at the irradiated site was corrected for the background signal from an equivalent area of the unirradiated part of the same nucleus for all techniques (Fig. 1e, right chart).

### Validation of the *in situ* fractionation protocol with GFP-tagged exogenous PARP-1 or its N-terminal DNA binding domain

**Figure 2:**
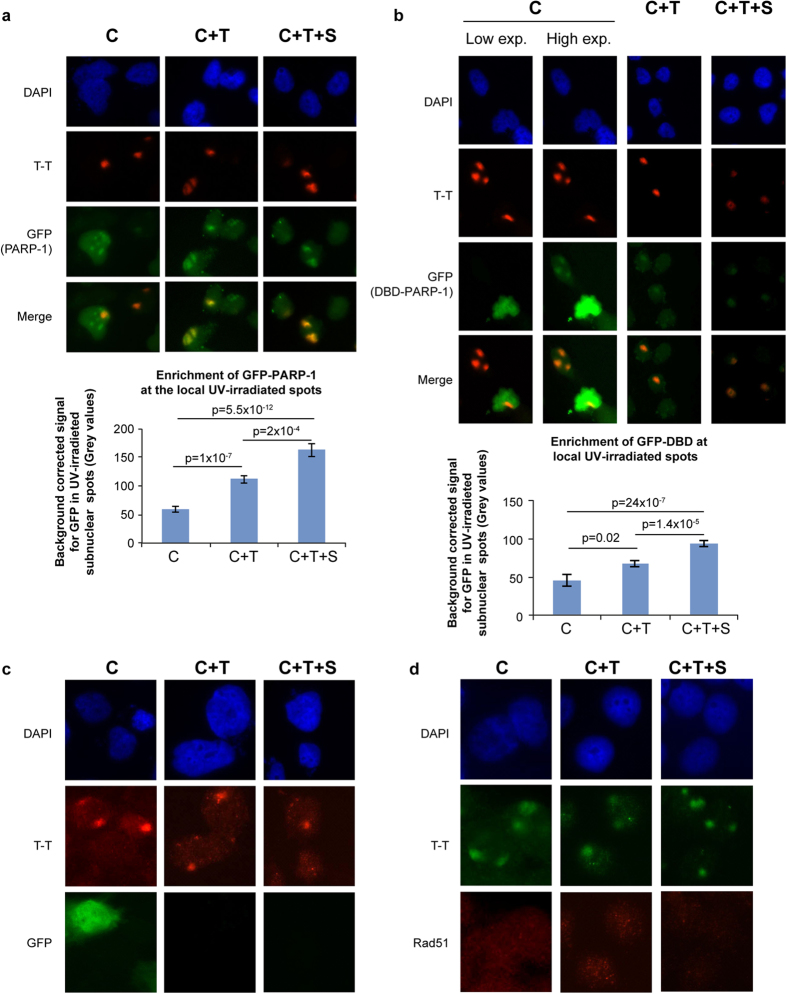
*In situ* fractionation improves detection of exogenous GFP-PARP-1 or its DNA binding domain at local UV-irradiated spots. **(a,b)** Recruitment of GFP-PARP-1 or its DBD to UV-induced T-T lesions. GMU6 cells were transfected with GFP- PARP-1 or GFP-DBD of PARP-1 for 24 h. The cells were locally irradiated and processed by C, C+T or C+T+S protocols. GFP-PARP-1 or GFP-DBD (green) and T-T (red) were visualized in DAPI-stained nuclei by immunofluorescence microscopy. The charts represent the quantification of GFP intensity for GFP- PARP-1 or GFP-DBD of PARP-1 at the T-T spots after background correction. (*n* = 80–150 spots, data points are mean ± s.e.). **(c,d)** Specificity of *in situ* extraction protocol: unrelated nuclear proteins (GFP and Rad51) do not colocalize with UV-damaged DNA. GMU6 cells were locally irradiated with 100 J/m^2^ UVC and subjected 10 min later to *in situ* fractionation using the three protocols. For panel-c, the cells were transfected with GFP expressing plasmid 24 h before irradiation and protein extraction. The cells were processed for immunofluorescence detection of GFP, Rad51 (green) and T-T (red). DAPI staining was carried out to define nuclei.

We compared the efficiency of each of the three protocols in revealing the recruitment of exogenous GFP-tagged PARP-1 to UV-induced T-T lesion in locally UV-irradiated cells (Fig. 2a). The C protocol was inefficient in revealing the co-localization of GFP signal with T-T spots especially in the cells expressing higher levels of exogenous PARP-1. The C+T and C+T+S protocols increasingly resolved this problem by removing “free” PARP-1, thus giving a background-corrected signal for GFP-PARP-1 at T-T lesion that was 1.9 and 2.7 times better than the C protocol, respectively (Fig. 2a, chart). The additional advantage was that this co-localization could be readily observed whether these cells initially expressed high or low levels of GFP-PARP-1.

The N-terminal DNA binding domain (DBD) of PARP-1 containing first two zinc fingers was shown to be sufficient for its recruitment to different types of DNA damages caused by laser irradiation of cells^20,21^. In the cells transiently transfected with GFP-DBD, the colocalization of DBD with T-T was evident only in low DBD-expressers, as shown in low and high exposure panels of C-protocol (Fig. 2b). The ability to discern co-localization of GFP-DBD to T-T lesion sites was significantly improved by 1.5 and 2.1 times with C+T protocol and C+T+S protocol, respectively (Fig. 2b, chart). Immunoblotting for GFP-DBD and endogenous untagged PARP-1 in control cell pellets in these extraction protocols revealed that the extent of removal of exogenous GFP- DBD at each step was similar to that of the endogenous cellular PARP-1 (Supplementary Fig. S1c). Our results show that the N-terminal DBD of PARP-1 is sufficient to recognize and bind to UVC-induced DNA damage.

To assess the specificity of the new protocol, we examined the status of UV-induced colocalization of unrelated proteins, such as the exogenous tag protein GFP (Fig. 2c) or the cellular DNA double strand break-repair protein Rad51 (Fig. 2d) at UV-lesion spots after processing with all three protocols. Although C+T and C+T+S protocols progressively removed both of these proteins from the cells, neither GFP nor Rad51 colocalized with UV-induced T-T lesions. Thus, our results show that the C+T+S protocol does not cause an artifact of random colocalization of unrelated proteins with UV-damaged DNA. This simple yet selective *in situ* fractionation protocol to reveal PARP-1 at UV-lesion site would be useful in studying other NER-related roles of PARP-1 with or without its catalytic activation at the site of UV-damaged DNA.

### An oligo with defined UV-lesion for restriction mapping of PARP-1

**Figure 3:**
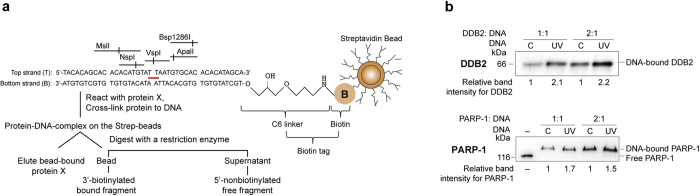
Strategy to study binding and footprint of proteins on UV-DNA. **(a)** The experimental design for determining the extent of binding of proteins to UV-DNA and analyses of protection of DNA during restriction digestion. The control and UV-DNA were immobilized on streptavidin beads via their biotin tag and reacted with purified PARP-1 or DDB2. The unreacted proteins were removed and bound proteins were cross-linked. The beads were then either analysed for bound-proteins by eluting the protein in Laemmli buffer, followed by SDS-PAGE, transfer and probe with specific antibodies or they were subjected to restriction digestion followed by analyses of the released DNA fragments on 10–15% native PAGE stained with gel red. **(b)** DDB2 and PARP-1 bind more to UV-DNA than control DNA. PARP-1 and DDB2 were reacted with 50 ng of control or UV-DNA at 1:1 or 1:2 (DNA:protein) molar ratios. The proteins were eluted from the beads, resolved on SDS-PAGE and analyzed by immunoblotting as shown in (**a**). The band intensities of protein bound to UV-DNA are shown as relative to signal for protein bound to control DNA.

To determine the exact footprint of PARP-1 at the UV-lesion site, we created biotin-tagged 40 mer oligonucleotides with or without a single defined UV-lesion surrounded by multiple unique restriction enzyme sites (Fig. 3a and Supplementary Fig. S2a). The UVC-irradiation of the top strand of the oligo, which has only one pair of adjacent Ts and no other adjacent pyrimidines (T or C), resulted largely in the formation of T-T rather than 6–4PP lesions (Supplementary Fig. S2b). The inability of restriction enzymes to digest through UV-induced CPD^22,23^ was exploited for purification of UV-DNA with VspI enzyme to remove all DNA molecules that did not form T-T at this site (Supplementary Fig. S2c). The biotin-tagged complementary strand for both control and UV-DNA allowed a common procedure for their immobilization to streptavidin beads (Fig. 3a). We reasoned that any protein bound at or around the UV-lesion site would prevent the restriction enzyme from digesting the DNA at that site; and thus decrease the quantity of non-biotinylated 5′-restriction fragment released from bead-bound DNA into the supernatant. This model allowed us to compare the extent of binding of proteins to control versus UV-DNA and also provide a non-isotopic method to footprint proteins on UV-DNA.

### PARP-1 and DDB2 bind more to UV-DNA than control DNA

We had shown in the cells and *in vitro* that PARP-1 not only binds to UV-damaged DNA^11^, but also interacts with DDB2 in the vicinity of UV-induced DNA lesions^7^. Using the model oligo described in Fig. 3a, we examined the extent of binding of PARP-1 and DDB2 to control and UV-DNA at two different molar ratios of protein : DNA (Fig. 3b). The 2.1−2.2× fold higher binding of DDB2 to UV-DNA than the control-DNA at these two molar ratios is in agreement with a previous report^24^. The binding of PARP-1 to UV-DNA was also 1.5−1.7× fold more than the control-DNA (Fig. 3b). We confirmed that PARP-1 did not bind to the beads per se unless DNA was attached to it (Supplementary Fig. S3a: left panel). To determine the site of PARP-1 binding to the control DNA without UV-lesion, we digested PARP-1-bound control DNA with VspI or NspI and noted that PARP-1 was attached more to the bead-bound 3′-end than to the 5′-end that is released after the restriction digestion (Supplementary Fig. S3a: right panel). This could be due to the linker attached biotin providing a pseudo-overhang at the 3′-end unlike blunt 5′-end, because PARP-1 has higher affinity for overhangs as compared to the blunt ends of DNA^25^. However, since the same 3′ and 5′-ends exist in both control and UV-DNA, any increase in PARP-1 binding to UV-DNA as compared to control-DNA must be due to the interaction of PARP-1 with UV-lesion, which we footprinted using a series of restriction enzymes that digest on either side of the lesion.

### Restriction protection profile of PARP-1 is distinct from that of DDB2 on either side of the T-T lesion

**Figure 4:**
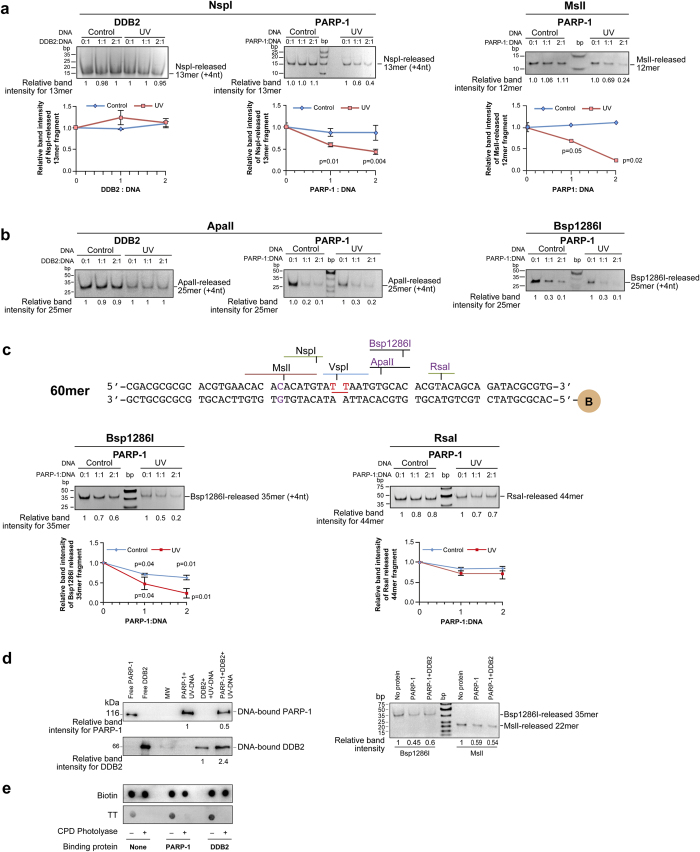
Footprinting of PARP-1 and DDB2 at UV-lesion site. **(a)** Restriction mapping of proteins on the 5′of the UV-lesion on 40mer DNA. 100 ng of bead-bound control or UV-DNA were reacted with DDB2 or PARP-1 at different DNA: protein ratios and digested at 37 °C with NspI (30 min) or MslI (15 min). The released 5′-fragments were resolved on 15% native PAGE and band intensities were measured. Each data point derived from three independent experiments represents mean ± s.d. for relative band intensity from three experiments for the fragment released from protein-bound versus protein-free DNA, with *P* values shown in the chart. **(b)** Mapping of proteins on the 3′-side of the UV-lesion on 40mer DNA. The protein-bound DNA was digested with ApalI (60 min) and Bsp1286I (20 min), and released 5′-fragments were resolved on 12% native PAGE. The data derived from two independent experiments is represented in the chart as described in panel-a. **(c)** *Top panel*-Structure of 60mer oligo with defined UV-damage. The 60mer oligo sequence was based on 40mer oligo but with a new RsaI site near 3′-end. *Bottom panels-* Restriction mapping of proteins on the 3′-side of the UV-lesion on 60mer DNA. The protein-bound DNA was digested with Bsp1286I (20 min) and RsaI (30 min) and released 5′-fragments were analysed by 12% native PAGE. The data derived from three independent experiments is represented in the chart as described in panel-a. **(d)** Simultaneous binding and footprint of DDB2 and PARP-1 on 60mer UV-DNA. PARP-1 and DDB2 were reacted either separately or together with bead-bound UV-DNA (50 ng), at 2:1 molar ratio of protein:DNA. The proteins bound to the beads were detected by immunoblotting (left panels), and footprint of proteins on DNA was examined by restriction digestion with Bsp1286I and Msll (right panels). **(e)** Repair of T-T by CPD photolyase despite binding of DDB2 or PARP-1 to UV-DNA. Bead-bound 40mer UV-DNA was reacted (or not) with DDB2 or PARP-1, and subjected to repair (or not) by CPD photolyase. The DNA was eluted and immunodot-blotted for T-T. The data represents identical results obtained in three independent experiments.

We established the optimal amount of DNA required in the assay for detection of DNA fragments released after digestion with restriction enzyme (Supplementary Fig. S3b) and also confirmed that both the control and UV-DNA without bound protein could be digested by all the restriction enzymes used in our footprinting assays (Supplementary Fig. S3c,d). Thus, any restriction protection offered to DNA after reaction with protein could be attributed to the footprint of the protein. During the restriction digestion by NspI and MslI that recognize sequences from −2 to −12nt on the 5′-side of the T-T lesion, PARP-1 offered more protection to UV-DNA than control DNA, as seen from a significant PARP-1 dose-dependent decrease in the corresponding 5′-fragments released by these enzymes (Fig. 4a). Thus, the footprint of PARP-1 on UV-DNA extended from 2–12nt upstream of the lesion site. In contrast, DDB2 failed to protect UV-DNA against NspI (−2 to −7 nt), indicating that its footprint stays within 2nt on the 5′-side of T-T, as reported earlier^26^.

PARP-1 has been shown to have a bilateral footprint of 7nt on either side of DNA single strand breaks^27^. Therefore, we compared the protections offered by PARP-1 and DDB2 against the restriction enzymes ApalI and Bsp1286I that target from 3 to 9nt on the 3′-side of the T-T lesion. The DDB2 did not offer any protection to control or UV-DNA against ApalI (Fig. 4b), indicating that its footprint does not exceed beyond 3nt on 3′-side of T-T, as reported earlier^26^. In contrast, PARP-1 offered a strong but equal protection to both UV and control oligos against both the enzymes, possibly due to PARP-1 bound to 3′-ends of both types of DNA, as noted earlier (Supplementary Fig. S3a: right panel). Thus using 40mer oligo, it was difficult to discern additional protection, if any, offered by PARP-1 that is bound at or near T-T site from the protection offered by PARP-1 that is bound to the 3′-end of the oligo.

To resolve this issue, we extended the size of 40 mer oligo on either ends to create a 60 mer, in which 3′-end was significantly separated from these two restriction sites and added a new RsaI restriction site at +11 nt from T-T (Fig. 4c, top panel). PARP-1 significantly protected 60mer UV-DNA against digestion by Bsp1286I as compared to control DNA, confirming its footprint up to +9nt from T-T lesion. Moreover, the lack of any additional protection by PARP-1 to UV-DNA against digestion by RsaI defined that PARP-1 footprint does not reach up to +11nt from T-T site (Fig. 4c). Thus using restriction-mapping technique on our model oligo, we show that PARP-1 bound at or near the T-T lesion extends asymmetric bilateral protection against restriction digestion from −12 to +9nt around the lesion.

Next, we examined whether PARP-1 and DDB2 can simultaneously bind to UV-DNA and whether DDB2 bound at the T-T lesion would alter the footprint of PARP-1 around the lesion. Using 60mer oligo, we noted that when incubated together, both DDB2 and PARP-1 could bind to UV-DNA (Fig. 4d, left panel). We observed in two independent experiments that the presence of PARP-1 increased the binding of DDB2 to UV-DNA, whereas that of DDB2 reduced the binding of PARP-1. However, the presence of DDB2 did not affect the restriction footprint of PARP-1 on UV-DNA, because PARP-1 offered identical protection to UV-DNA against restriction digestion on either side of the lesion site by MslI and Bsp1286I in the absence or the presence of DDB2 (Fig. 4d, right panel). Thus, the footprint of PARP-1 around the lesion was not compromised in the presence of DDB2 whereas DDB2 was more stabilized in the presence of PARP-1.

### No effect of PARP-1 and DDB2 binding on CPD-photolyase mediated repair of T-T in UV-DNA

Unlike restriction enzymes that cleave the DNA in the sugar phosphate backbone, the CPD photolyase directly removes the cross-linking of adjacent pyrimidines in the CPD photolesions such as T-T^28^. Structural studies have revealed that DDB2 has a protein fold that flips out and maintains contact with T-T^26^, and our results indicate that PARP-1 also remains in the vicinity of T-T lesion. Hence, we examined whether binding of DDB2 or PARP-1 to UV-DNA could influence the repair of T-T by CPD photolyase (Fig. 4e). The immunodot-blot of DNA with or without photolyase treatment revealed that binding of DDB2 or PARP-1 to DNA could not inhibit the ability of photolyase to repair the T-T lesions, indicating that these two proteins do not exclude other repair proteins from accessing the lesion. This is also in agreement with a previous report that binding of DDB2 to UV-DNA does not prevent CPD photolyase from repairing the UV-lesion^29^.

### Catalytic activation of PARP-1 is stronger with 6–4PP than T-T lesion

**Figure 5:**
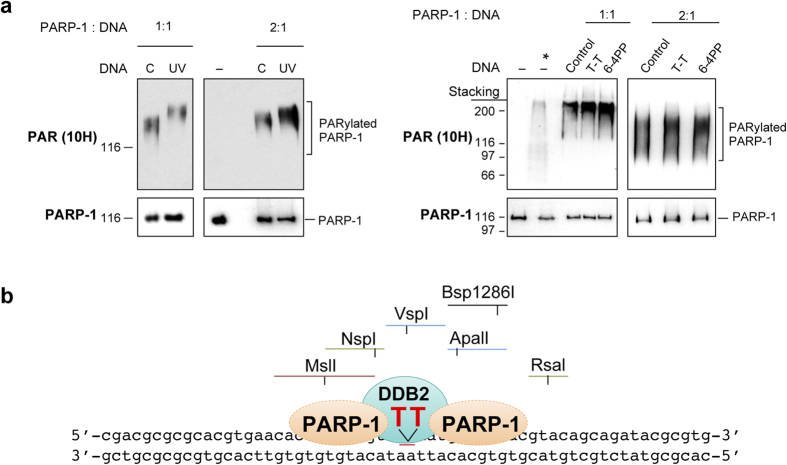
Catalytic activation of PARP-1 with defined UV-damaged DNA and a model for footprint of PARP-1 and DDB2 on UV-lesion site. (**a)** Stimulation of catalytic activity of PARP-1 by various defined UV-damaged DNA *in vitro*. PARP-1 activation assay was performed using 40 mer control or UV-DNA (left panel) or using 24mer DNA with no damage (control) or with a single defined T-T and 6–4PP (right panel) at 1:1 or 1:2 molar ratio of DNA:protein. After resolution on SDS-PAGE, immunoprobing for PARP-1 and PARylated PARP-1 (10H antibody) was carried out. The *refers to the cell extract containing H_2_O_2_-activated PARP-1. Panel represents one of three identical experiments. **(b)** Model for binding of PARP-1 and DDB2 to the UV-lesion site on 60mer oligo (see Results and Discussion section for details).

We used the model oligos for further characterization of the interaction of PARP-1 with UV-damaged DNA. Since the catalytic activation of PARP-1 is more with damaged DNA than with undamaged DNA^14^, we examined the extent of activation of PARP-1 with 40mer control or UV-oligo *in vitro* as an additional indicator of the extent of binding of PARP-1 to these DNA. A stronger PARP-1 activation with UV-DNA was observed as compared to control-DNA at two different molar ratios of PARP-1 to DNA (Fig. 5a, left panel). Thus the binding of PARP-1 to UV-lesion caused stronger stimulation of its catalytic activity as compared to its binding to either the 3′ or 5′-ends of both the control and UV-DNA. To compare the capacity of different UV-induced direct damages for activation of PARP-1, we assessed the efficacy of 24mer oligo containing a single chemically synthesized T-T or 6–4PP lesion^30^ in the PARP-1 activation assay *in vitro* (Fig. 5a, right panel). Both the DNA containing defined UV-lesions were stronger activator of PARP-1 than the 24mer control-DNA at two different molar ratios of PARP-1 to DNA. Moreover, 6–4PP, which is known to cause a higher degree of helical distortion, was a stronger stimulator of the catalytic activity of PARP-1 than T-T.

### Model for the interaction of PARP-1 and DDB2 with UV-damaged DNA

Based on the current results of *in situ* visualization and footprinting of PARP-1 and DDB2 at UV-induced DNA lesion site, we propose that DDB2 attaches directly at the UV-lesion site whereas PARP-1 makes an asymmetric bilateral contact from −12 to +9nt around the lesion (Fig. 5b). This footprint is compatible with either one or two PARP-1 molecules enveloping around the UV-lesion, similar to the reported binding of either one^31^ or two^21^ PARP-1 molecules at the site of DNA strand breaks. The N-terminal DNA binding domain of PARP-1 that is known to be recruited to DNA strand breaks^20^ was also recruited to UV-lesions without strand breaks, indicating more general property of this domain of PARP-1 to bind to different types of DNA damages. Our model is also consistent with previously reported interactions of DDB2 and PARP-1 at the UV-lesion site^5,7^. We have earlier shown that PARP-1 and DDB2 co-immunoprecipitate at the UV-damaged chromatin in the presence of ethidium bromide, indicating their direct interaction on the same DNA strand^7^. Here, we confirm that PARP-1 and DDB2 can co-exist at the UV-lesion site and the presence of DDB2 does not alter the footprint of PARP-1 around the lesion. Our results do not exclude the possibility that PARP-1 footprint may vary when the damaged DNA is in the context of chromatin or when there are multiple UV-lesions in close proximity. We had earlier shown that PARP-1 is weakly activated by UV in the DDB2-deficient XP-E cells, but introduction of DDB2 in these cells strongly stimulates PARP-1 catalytic activity in response to UV^7^. Moreover, we had also observed that DDB2 stimulates catalytic activity of PARP-1 *in vitro* with UV-damaged DNA that largely contains T-T lesions^7^, and here we show that PARP-1 activation was much stronger with 6–4PP than with T-T. Since the 6–4PP by itself causes larger distortion of DNA as compared to T-T, whereas the binding of DDB2 to T-T increases the distortion of DNA^26^, collectively these results indicate that the extent of DNA distortion could be the determinant for PARP-1 activation with UV-damaged DNA. Our results show that PARP-1 and DDB2 do not prevent access to the lesion site by other repair proteins such as CPD photolyase, and may even be more flexible and accommodating in the *in vivo* conditions as compared to our *in vitro* condition in which damaged DNA is anchored to beads and interacting proteins are cross-linked to the DNA. Together, these two novel assays will open new avenues to study the ever-expanding roles of PARP-1 in NER and housekeeping functions in which PARP-1 shows subtle interactions with DNA without strand breaks.

## Material and Methods

### Antibodies

For Western blotting: Monoclonal 6–4PP (KTM50) and T-T (KTM53) (1:5,000), polyclonal PARP-1 (1:5,000), PARP-1 monoclonal (F2, 1:500), GFP monoclonal (1:5,000), DDB2 anti goat (1:500), PAR monoclonal (10H, 1:1,000), HRP-conjugated secondary antibodies (1:2,500). For immunocytology: T-T (KTM53, 1:2,000), polyclonal PARP-1 (1:5,000), PARP-1 monoclonal F2 (1:500), PARP-1 monoclonal C2-10 (1:500), F1-23 monoclonal (1:500), DDB2 anti goat (1:200), Rad51 polyclonal (1:500) and secondary fluorescent antibodies (1:500).

### Cell culture

Cells were grown in 5% humidified incubator at 37 °C in αMEM medium supplemented with 10% FBS, penicillin and streptomycin and 200 μg/ml hygromycin. The creation of GM637-derived PARP-1 replete GMU6 cells were described earlier^32^.

### Expression Plasmids

The pGFP-hPARP-1 was a gift from V. Schreiber, pGFP-DBD of PARP-1 was generated by subcloning the 0.7 kb SacI (blunted) and HindIII-fragment of PARP31 cDNA into NheI (blunt)/HindIII sites of pEGFP-N1 plasmid.

### Transfection and UVC-irradiation

GMU6 cells were transfected with pEGFP-hPARP-1, pEGFP-DBD-PARP-1 and pEGFP-N1 plasmid using Turbofect reagent. After 24 h, cells were irradiated for global UVC (10 J/m^2^) or through polycarbonate filter with 5 μm pores for local (100 J/m^2^) UVC^11^.

### Indirect immunofluorescence detection *in situ*

The cells adherent on the coverslip were processed by one of the three protocols: *C protocol:* After washing with CSK buffer (100 mM NaCl, 300 mM sucrose, 10 mM PIPES pH 6.8, 3 mM MgCl_2_, 1 mM EGTA), cells were fixed with 3% formaldehyde (10 min, ambient temperature), rinsed with PBS, permeabilized with 100% methanol (−30 °C, 10 min), rinsed with PBS, blocked for 30 min with 5% BSA in PBS-0.1% Triton-X-100 followed by reaction with primary antibodies given below. *C*+*T protocol:* Cells were washed twice with CSK buffer, extracted with 0.5% Triton in CSK buffer for 8 min at ambient temperature, fixed and blocked as in C protocol. *C*+*T*+*S protocol:* After C+T protocol fixation step, cells were washed and extracted with 0.5% Triton in high salt CSK buffer (420 mM NaCl, 300 mM sucrose, 10 mM PIPES pH 6.8, 3 mM MgCl_2_, 1 mM EGTA) for 20 min on ice. After DNA denaturation in 0.07 N NaOH for 8 min at ambient temperature, immunoprobing was carried out for all three protocols as follows: The cells were incubated for 1 h with primary antibodies in the blocking buffer, washed with PBS containing 0.1% Tween 20 and incubated for 30 min with Alexa 488 or 594-linked secondary antibodies. After washing with PBS-0.1% Tween 20, coverslips were incubated in PBS-0.25 μg/ml DAPI for 5 min and mounted with ProLong Gold Antifade. Images were captured with Zeiss Axiovert 200 and AxioCam MRm and the brightness and contrast were uniformly adjusted across the panels with Photoshop CS5.5. The fluorescent intensity of PARP-1 at the irradiated spots was analyzed with AxioVision 4.7 and corrected for background signal for similar area in the unirradiated zone of nucleus, where specified.

### Statistical analyses for immunocytology

Data for intensity of at least 100 foci from three independent experiments were pooled to create mean + s.e., and subjected to the unpaired two-tailed t-tests to determine the significance of difference. The significant *P*-values <0.05 are stated in the charts.

### Oligos

The ss-24mer oligos with T-T or 6–4PP were chemically synthesized and hybridized with the complementary ss-24mer to get 24mer ds oligo with T-T or 6–4PP lesions. The creation of 40 or 60mer oligos are described in Figs. 3a, 4c, and Supplementary Fig. S2a.

### VspI-purification of 25 kJ/m^2^ UVC irradiated oligo

The UVC irradiated 40mer or 60mer ds oligos were digested with VspI for 1 h at 37 °C, followed by VspI-inactivation at 65 °C for 5 min. The VspI-resistant 40 or 60mer UV-DNA were separated on 12% PAGE from the digested fragments of DNA without UV-damage, cut from the gel and eluted with PAGE elution buffer (0.5 M ammonium acetate, 10 mM magnesium acetate and 1 mM EDTA) overnight at 37 °C. The eluted UV-DNA were cleaned by passage through ULTRAFREE–DA filter units, concentrated in Microcon YM-10 followed by purification in Zymo Research oligo clean and concentrator columns.

### The protein-DNA interactions, restriction mapping and CPD photolyase assays for bead-bound oligos with proteins

The control or UV-DNA with biotin tag was immobilized on Dynabeads MyOne streptavidin T1. (i) The binding of PARP-1 or DDB2 to control or UV-oligos immobilized on the magnetic streptavidin beads: The bead-bound oligos (50–100 ng) were reacted with PARP-1 or DDB2 at 1:1 or 1:2 molar ratio. PARP-1 was reacted at 25 °C for 15 min in 10 μL of Na-PO_4_ reaction mixture (20 mM of Na-PO_4_ buffer pH 7.4, 5 mM MgCl_2_, 150 mM NaCl, 5% glycerol, 1 mM DTT, 0.01% Triton, 20 μM Zn-acetate and 1 × protease inhibitor). DDB2 was reacted at 25 °C for 30 min in Tris reaction mixture (100 mM Tris buffer pH 8.0, 10 mM MgCl_2_, 10% glycerol, 150 mM NaCl, 1.5 mM DTT and 1× protease inhibitor). The simultaneous binding of PARP-1 and DDB2 was carried out in Tris reaction mixture for 15 min at 25 °C. The unbound proteins were removed and the bound proteins were crosslinked to oligo with 1% formalin in Na-PO_4_ reaction mixture for 10 min at ambient temperature. After quenching formalin with 250 mM Tris-HCl pH 8.0, the beads were washed twice with Tris-reaction mixture and subjected to following steps.

(ii) Assessment of binding of proteins with DNA: The proteins that were attached to bead-bound DNA were extracted with Laemmli buffer at 95 °C for 10 min. The eluted proteins were resolved on SDS-PAGE for immunoblotting of the protein. The band quantification for immunoblots was carried out with ChemiGenius 2 using SynGene software.

(iii) Restriction protection assay of protein-bound oligos: For the restriction protection assay, the magnetic streptavidin beads bound control or UV-DNA with or without bound protein were digested with the specified Fast-digest or CutSmart restriction enzyme in 10 μL reaction buffer at 37 °C for specified time. The DNA fragments released in the supernatant were resolved on 10–15% native PAGE and stained with gel red for identification and quantification with ChemiGenius2 using SynGene software. The relative band intensities were derived by comparing the intensities of the fragments released from the protein-bound DNA with that from protein-free DNA. The significance was calculated by unpaired two-tailed t-test and the *P* values <0.05 were considered significant.

(iv) CPD photolyase repair assays: The bead-bound UV-DNA with or without attached proteins was split into two aliquots; one subjected to repair by CPD photolyase and the other was mock-treated. The CPD repair was carried out in 20 μL CPD photolyase binding mix (20 mM Tris buffer pH 7.5, 1 mM DTT, 0.2 mg/ml BSA, 125 mM NaCl) with 0.2 μL Oryza sativa CPD photolyase and incubated for 15 min in dark at ambient temperature, followed by exposure for 15 min to UVA (Spectrolinker XL-1500, 363 nm, 15 watts). DNA was eluted with 95% formamide, 10 mM EDTA pH 8.0 for 5 min at 95 °C, dot-blotted on the Hybond N^+^ and probed for T-T.

### PARP-1 activation assay *in vitro*

In a 10 μL reaction mixture containing 100 mM Tris-HCl pH 8.0, 10 mM MgCl_2_, 10% glycerol, 1.5 mM DTT, 1× protease inhibitor and 10 μM NAD, purified PARP-1 was reacted at 25 °C for 30 min with specified DNA for each reaction. After adding an equal volume of 2× Laemmli buffer, samples were resolved on SDS-PAGE and immunoblotted for PAR (10H) and PARP-1 ^14^.

### Immuno-Dot-blot

DNA samples were heated at 95 °C for 5 min, chilled on ice for 5 min and adjusted to final concentration of 6× SSC. Samples were dot-blotted on Hybond N+ membrane, baked at 80 °C for 1–2 h and processed for antibody probing.

## Additional Information

**How to cite this article**: Purohit, N. K. *et al.* Characterization of the interactions of PARP-1 with UV-damaged DNA *in vivo* and *in vitro*. *Sci. Rep.* **6**, 19020; doi: 10.1038/srep19020 (2016).

## Supplementary Material

Supplementary Dataset 1
