# Supplementary material for: Characterization of the interactions of PARP-1 with UV-damaged DNA in vivo and in vitro
Source: Sci Rep. 2016 Jan 12;6:19020. doi: 10.1038/srep19020 (PMC4709520; doi:10.1038/srep19020)
Supplement: Supplementary Dataset 1 [file srep19020-s1.doc]

**Characterization of the interactions of PARP-1 with UV-damaged DNA in vivo and in vitro**

Nupur K. Purohit^1#^, Mihaela Robu^1#^, Rashmi G. Shah^1#^, Nicholas E. Geacintov^2^ and

Girish M. Shah^1*^

**Supplementary Fig. S1**


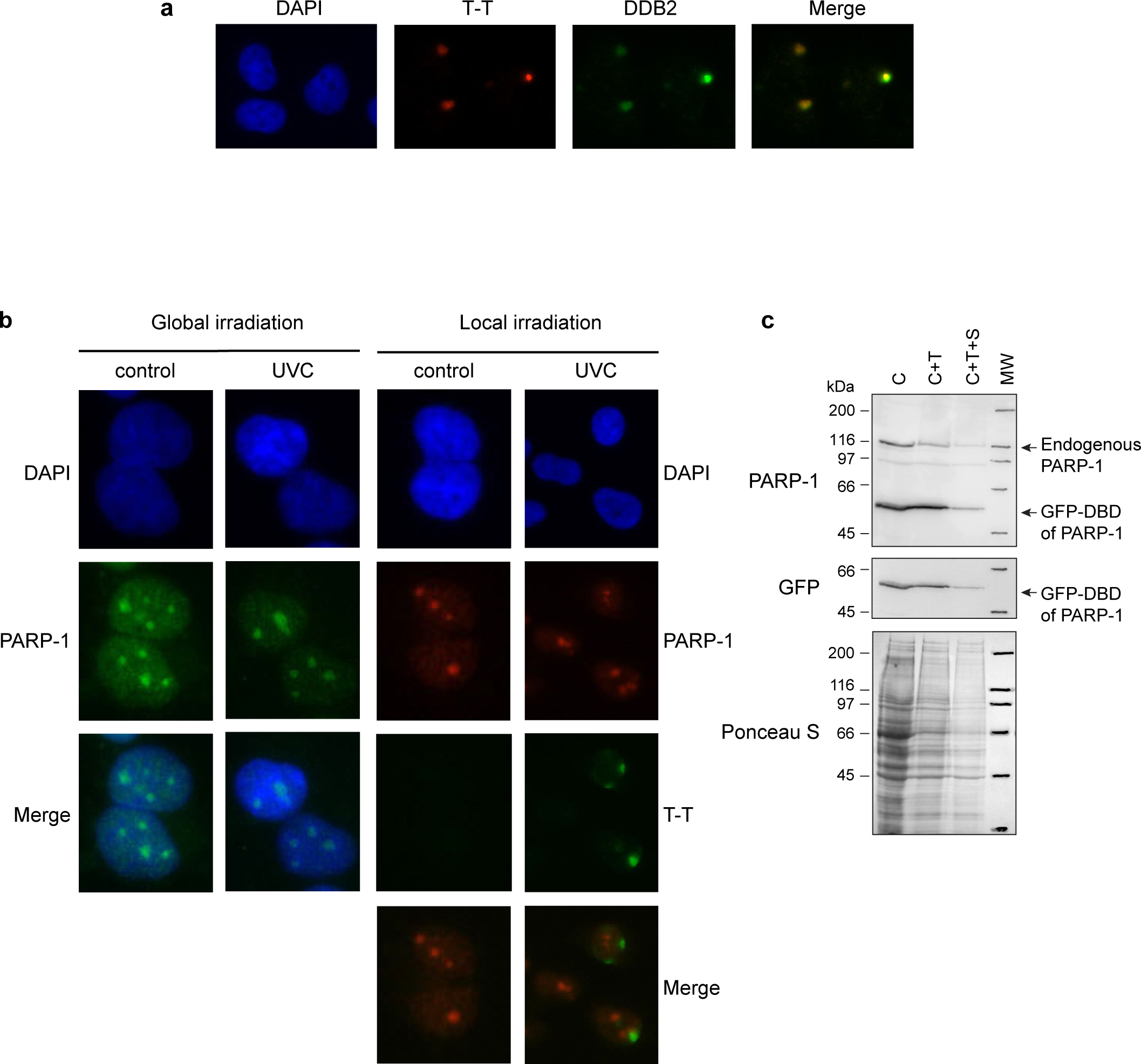


**Supplementary Fig. S1. (a)** DDB2 colocalizes with T-T spots. GMU6 cells locally irradiated with 100 J/m^2^ UVC were probed after 10 min for DDB2 (green) and T-T (red) in DAPI stained cells. **(b)** PARP-1 spots after formaldehyde-Triton protocol do not colocalize with T-T. Human skin fibroblasts growing on coverslips were exposed either to global (10 J/m^2^) or local (100 J/m^2^) irradiation with UVC and 10 min later fixed with formaldehyde for 10 min followed by 5 min permeabilization with 0.5 % Triton. The globally irradiated cells were probed for PARP-1 (green) and the locally irradiated cells for PARP-1 (red) and T-T (green). The nuclear DNA was stained with DAPI. **(c)** Verification of the in situ protocol for extraction of GFP-DBD and endogenous PARP-1 in control cells by Western blot. The GMU6 cells were transfected with GFP-DBD of PARP-1 and 24 h later extracted with C, C+T and C+T+S buffers. The cell pellets from equivalent number of cells for each of the three protocols were immunoblotted for GFP and PARP-1. Ponceau S staining reflected the residual protein content in cell pellets at the end of each protocol.

**Supplementary Fig. S2**

**
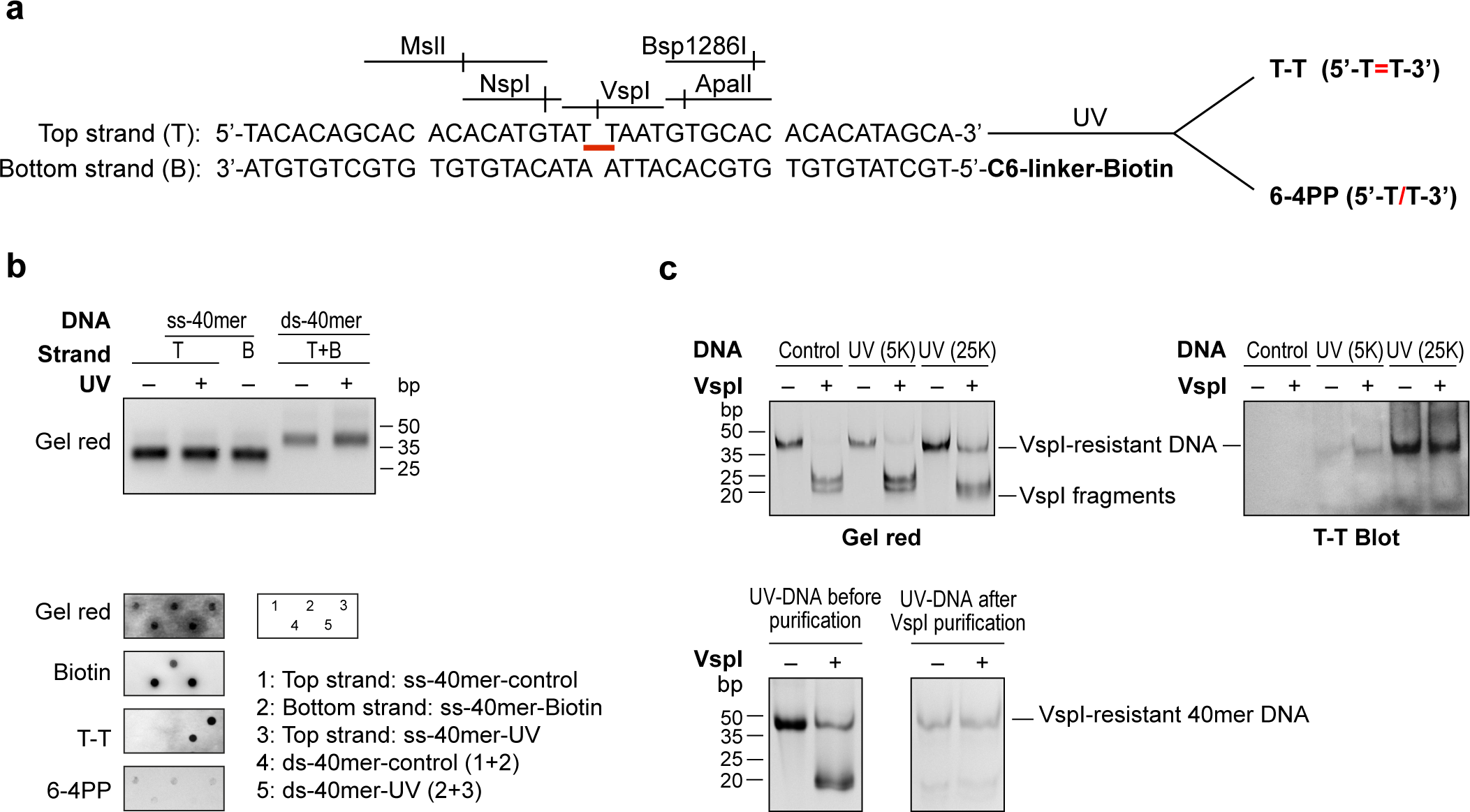
**

**Supplementary Fig. S2.** Preparation and characterization of double stranded (ds) 40mer DNA. **(a)** Construction of biotinylated ds 40mer with and without defined T-T*.* The top single strand (ss) 40mer sequence was designed to carry only one adjacent pair of Ts, and no other pair of pyrimidines, i.e., T or C. The complementary bottom ss 40mer contained a biotin tag at its 5’-end attached via a 6-carbon linker chain. For UV-DNA with defined T-T, the top strand was irradiated in TE at 25,000 J/m^2^ UVC at the fluence rate of 51.2 J/m^2^/sec in Spectrolinker XL1000. Where specified, top strand was also irradiated with lower UVC-dose (5,000 J/m^2^) at the fluence rate of 10 J/m^2^/sec. The control and UV-irradiated top ss oligos were annealed with biotin tagged complementary bottom strand to form control and UV ds 40mer oligos, respectively. **(b)** *Top panel*-The ss 40mer and ds 40mer were verified on agarose gel. *Bottom panel*- 25 ng of the individual ss and the resulting ds 40mer were spotted on charged nylon membrane, and probed for T-T, 6-4PP, biotin and stained with gel red (to indicate the loading of DNA). **(c)** Enrichment of 40mer UV-DNA using VspI*.* Top left panel- Control, 5,000 and 25,000 J/m^2^ UVC irradiated DNA were digested (37˚ C for 1 hr) with VspI (or undigested) and resolved on 12 % native PAGE. Top right panel- Same as top left, but the native PAGE resolved DNA was transferred on charged nylon membrane for 75 min at 0.2 A (45-60V) and probed for T-T. *Bottom left and right panel*-Purification of T-T containing DNA using VspI digestion. The undigested and VspI-digested 25,000 J/m^2^ UV-DNA were run on 12 % native PAGE before (bottom left) and after purification (bottom right).

**Supplementary Fig. S3**

**
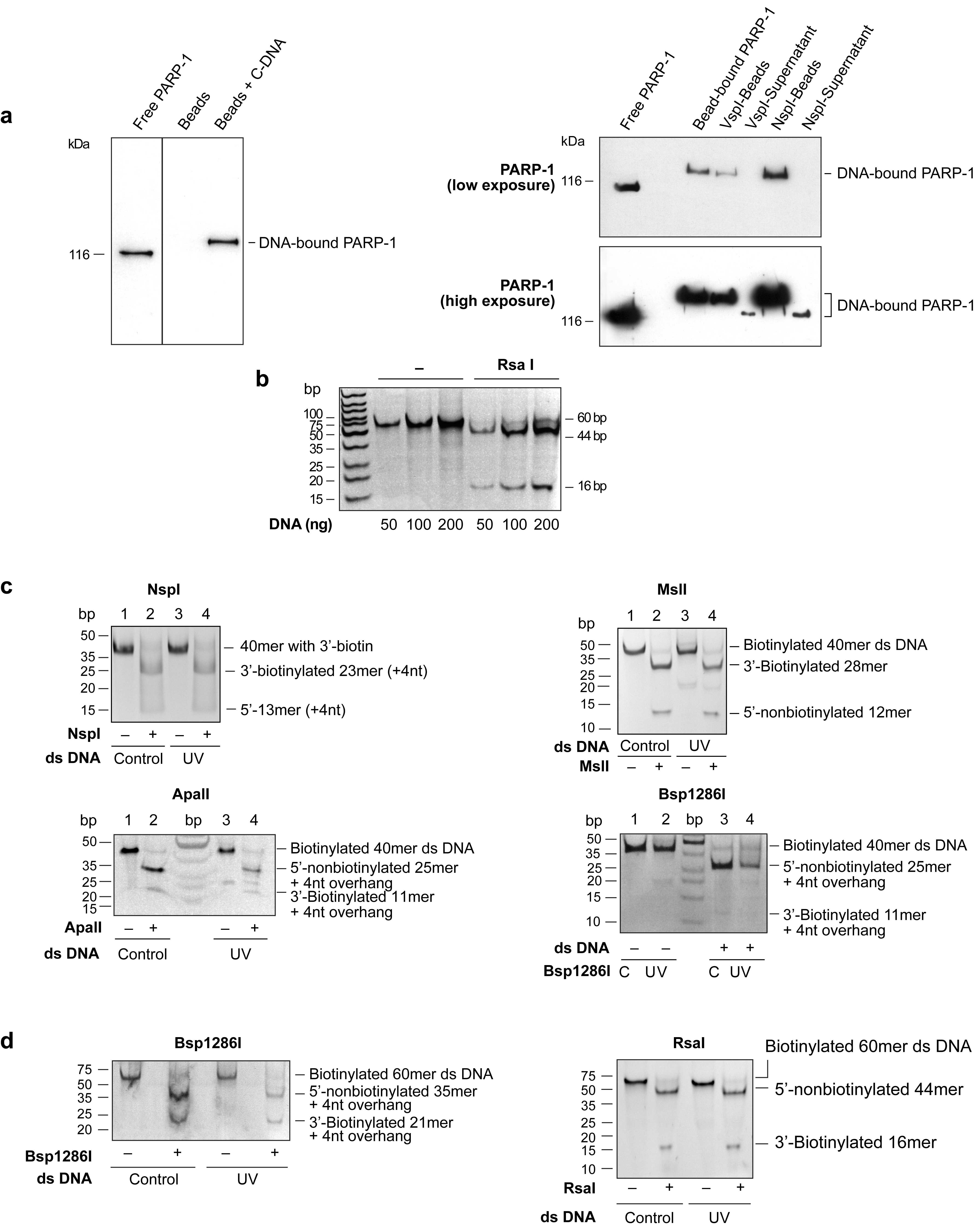
**

**Supplementary Fig. S3.** **Optimization of protein binding and restriction assays with control and UV-DNA**. **(a)** PARP-1 binds to bead-bound 40mer control DNA mainly at 3’-end of oligo and does not bind to streptavidin beads per se. *Left panel*- PARP-1 was reacted with either free streptavidin beads or beads bound to control DNA. The bound-protein was immunoblotted for PARP-1. *Right panel*- Bead-bound control DNA was reacted with PARP-1 and the reaction mixture divided in three equal aliquots. One aliquot was not reacted with any restriction enzyme (bead-bound PARP-1) and other two aliquots were digested with VspI or NspI. Immunoblotting was carried out to detect PARP-1 that was attached to the bead-bound undigested 40mer, the 5’-restriction fragment of DNA released in the supernatant and 3’-restriction fragment of DNA bound to the beads. **(b)** Optimization of DNA concentration in restriction digestion assay. The 50-200 ng control 60mer DNA were digested with RsaI and the resulting 3’ and 5’ DNA fragments were resolved on 15 % native PAGE and visualized using gel red. **(c)** Digestion of protein-free 40mer control or UV-DNA with different restriction enzymes. Both DNA were digested at 37˚ C with NspI for 30 min (top left panel), MslI for 15 min (top right panel), ApalI for 1h (bottom left panel) and Bsp1286I for 20 min (bottom right panel). The DNA or its fragments recovered from undigested and enzyme digested samples were resolved on native PAGE prior to detection with gel red. **(d)** Digestion of protein-free 60mer control or UV-DNA with different restriction enzymes. Both the DNA were digested at 37˚ C with Bsp1286I for 20 min (left panel) or RsaI for 30 min (right panel), followed by detection of DNA or its fragments as described above.
